# Thermal Conductivity of Diamond Composites

**DOI:** 10.3390/ma2042467

**Published:** 2009-12-21

**Authors:** Sergey V. Kidalov, Fedor M. Shakhov

**Affiliations:** Ioffe Physical-Technical Institute of the Russian Academy of Science, 26 Polytekhnicheskaya st., Saint-Petersburg, 194021, Russia; E-Mail: Fedor.Shakhov@mail.ioffe.ru (F.M.S.)

**Keywords:** diamond, thermal conductivity, heat sink, composite, nanodiamond, high pressures, infiltration, spark plasma sintering

## Abstract

A major problem challenging specialists in present-day materials sciences is the development of compact, cheap to fabricate heat sinks for electronic devices, primarily for computer processors, semiconductor lasers, high-power microchips, and electronics components. The materials currently used for heat sinks of such devices are aluminum and copper, with thermal conductivities of about 250 W/(m·K) and 400 W/(m·K), respectively. Significantly, the thermal expansion coefficient of metals differs markedly from those of the materials employed in semiconductor electronics (mostly silicon); one should add here the low electrical resistivity metals possess. By contrast, natural single-crystal diamond is known to feature the highest thermal conductivity of all the bulk materials studied thus far, as high as 2,200 W/(m·K). Needless to say, it cannot be applied in heat removal technology because of high cost. Recently, SiC- and AlN-based ceramics have started enjoying wide use as heat sink materials; the thermal conductivity of such composites, however, is inferior to that of metals by nearly a factor two. This prompts a challenging scientific problem to develop diamond-based composites with thermal characteristics superior to those of aluminum and copper, adjustable thermal expansion coefficient, low electrical conductivity and a moderate cost, below that of the natural single-crystal diamond. The present review addresses this problem and appraises the results reached by now in studying the possibility of developing composites in diamond-containing systems with a view of obtaining materials with a high thermal conductivity.

## 1. Introduction

The main requirements placed by electronics engineers on heat sinks, formulated possibly not in the order of descending significance, are:

- high thermal conductivity
- low cost
- low electrical conductivity
- thermal expansion coefficient of the heat sink should be equal to that of the material to be cooled
- low loss tangent
- small weight

Most materials displaying high thermal conductivity, above 100 W/(m·K), at room temperature have diamond-like lattices; among them are diamond, cubic boron nitride and others, in particular, SiC, BeO, BP, AlN, BeS, GaN, Si, AlP, and GaP [1].

In applications where fairly high thermal conductivities combined with low electrical conductivities are of prime importance, SiC- and AlN-based ceramics are presently enjoying wide use. The progress in the development of technologies of production of nanosized crystals we are currently witnessing has generated a surge of interest in the investigation and application potential of nanomaterials, including aspects associated with fabrication of high thermal conductivity composites.

Having a high thermal conductivity [up to 2,200 W/(m·K)], diamond occupies a prominent place among the materials which offer promise for developing high-efficiency heat sinks for semiconductor lasers, high-frequency, high power transistors, optical amplifiers, power LEDs, integrated circuits, *etc*.

For composites designed to feature high thermal conductivity levels one ordinarily uses high-quality diamonds whose single crystals demonstrate a high value of this parameter, and nonferrous metals, such as copper λ = 380 W/(m·K), silver λ = 420 W/(m·K), and aluminum λ = 240 W/(m·K) [2]. An additional requirement imposed on high thermal conductivity composites is that its linear thermal expansion coefficient be equal to that of the material to be cooled—primarily that of semiconductors (Table 1).

Viewed in the scientific context, the problem consists essentially in that in metals heat transport is mediated by electrons, and that in diamond, by phonons. Therefore in composites with a metallic binder, energy is transferred between electrons and phonons. It is assumed that the diamond surface is coated by a very thin carbide layer which matches electrons with phonons. Already one of the first studies of the thermal resistance appearing at the diamond-metal interface demonstrated the significance of this aspect that governs the thermal properties of composites [3].

The solubility of carbon in Cu and Ag is measured in hundredths of a wt % [4]. The characteristics of the composite materials developed for use in heat sinks are dominated by the quality of the filler-binder boundary. The carbide-forming agents added to diamond and copper or silver are most frequently Ti, Cr, B, and Si.

Transition metal carbides (NbC, TiC, WC), nitrides, and borides may be called metalloceramics, because they combine electronic conduction with high hardness. It is in these layers on the diamond surface that electron-phonon interaction arises.

**Table 1:** Thermal conductivity and thermal expansion coefficients at room temperature of materials most widely employed in heat sink fabrication.

Material	Thermal conductivity, *λ* W/(m·K)	Thermal expansion coefficient, *α*·10^6^, К^-1^
Synthetic Diamond Ib (C)	1,000-2,200	1.1
Silicon (Si)	153	3.8
Silicon Carbide (SiC)	280-400	3.8
Aluminum Nitride (AlN)	285	4.3
Indium Phosphide (InP)	68	4.3
Gallium Arsenide (GaAs)	55	5.9
Copper (Cu)	380	16.5
Silver (Ag)	420	19.8
Aluminum (Al)	240	22

Indeed, it was shown [5] that by using an atomic alloy of copper with chromium (0.8 wt %) and Ib diamonds 177–210 µm in size (MDB4) it is possible to increase thermal conductivity up to 740 W/(m·K), to be contrasted with 200 W/(m·K) featured by a copper-diamond composite.

This idea is frequently used to improve the thermal conductivity of the diamond-binder boundary. Indeed, it was proposed [6] to apply titanium coating to particulate diamond from a NaCl melt at 1,000 °C, or NaCl+KCl+CaCl_2_ at 750 °C, an approach reportedly producing good results. The best agent to mediate diamond sintering at high pressures (6.5 GPa) and temperatures (1,600–1,800 °C) and produce composites with very good abrasive resistance was shown to be titanium carbide and TiC_0.6_ [7].

Ekimov *et al.* [8] demonstrated that Ti and Si present in diamond composites prepared at a pressure of 2 GPa, rather than being only a filler, form in actual fact a binder. In such composites, thermal conductivity depends only weakly on the size of diamond grains and reaches a value as high as 600 W/(m·K) when employed as a SiC-Si matrix.

The interest in composites fabricated by high-pressure diamond sintering stems primarily from the fact that they exhibit good strength and high abrasive properties and find wide application in industry. We have offered such a detailed description of the sintering parameters of microcrystalline particulate diamond in this review only to provide an adequate insight into the actual conditions and mechanisms operating in the sintering process, as well as to review the parameters of the present-day composites. The data pertaining to the sintering parameters were seen to exhibit considerable scatter, both in pressure and in temperature.

Papers dealing with sintering of detonation nanodiamonds (DND) at a high pressure are few in number. It appears already obvious, however, that microcrystalline diamonds and detonation nanodiamonds differ markedly in thermodynamic properties.

Viewed in the context of possible applications, of most interest would be the possibility of obtaining large (up to 600 µm in size) single crystals by a suitable thermal treatment of detonation nanodiamonds.

Basic science may be interested in investigation of the thermodynamic properties of DND, of the graphitization process, and sintering mechanisms giving rise to an increase of the nanodiamond coherent scattering regions, as well as of the bulk characteristics of the samples produced in DND sintering, such as density, thermal conductivity, strength, sonic velocity, *etc*.

A particular place should have been devoted in the review to analyzing the possibility of chemical modification of DND surface, because the latter can affect noticeably the properties of the samples obtained and their thermal conductivity; regrettably, this problem is beyond the scope of the present report and, thus, will not be dealt with here.

## 2. Methods Employed to Develop High Thermal-Conductivity Composite Materials

Among the most promising approaches to developing composite materials exhibiting high thermal conductivity are sintering at high-pressures and temperatures, infiltration of a melted metal into a matrix, and spark plasma sintering (SPS), a method that has appeared recently. Consider these methods in more detail.

### 2.1. High-pressure sintering

The definition “high pressure” assumes, as a rule, pressures above 1–1.5 GPa, *i.e.*, levels in excess of the ultimate strength of hardened steels. Materials employed in high-pressure applications are tungsten carbide-based hard alloys. Equipment enjoying the widest use in synthesis of superhard materials and their compaction are high-pressure chambers of the truncated hemisphere anvil and toroid types proposed by Vereshchagin, multi-plunger split-sphere and belt-type equipment. The pressures that can be reached with these chambers range from 4.5 GPa to 12 GPa. Photographs of a toroid-type chamber can be found in [8], and description of the split-sphere apparatus, for instance, in [9,10], with a variety of designs illustrated in [11]. The principal shortcoming of the high-pressure approach lies in the size of the chamber and, accordingly, that of the samples, and in heavy labor consumption. Toroid-type chambers are commonly used to fabricate cylindrical samples with a diameter and height of not over 3–5 mm. In most cases, high pressure is employed in fabrication of abrasive composites for manufacturing industry rather than of high thermal-conductivity materials. The high pressure method is instrumental in producing composites at temperatures above 1,000 °C, without the need for making the operating volume air-tight and providing means for its evacuation. Apart from this, by properly varying the pressure and maintaining a constant temperature, one can control phase transitions in such materials as, for instance, diamond, cubic boron nitride, *etc*.

### 2.2. Infiltration

This process consists essentially in infiltrating a liquid binder into the filler material. High thermal-conductivity composite materials are usually prepared with diamond particulates used as a filler, and metals (aluminum, copper, silver) for a binder. Infiltration can be driven by squeezing a metal into the filler mechanically (a process called squeeze casting) or through a conveniently applied pressure drop (gas pressure-assisted process). Because these metals do not wet diamond, carbide-forming agents are added to the melt to form a structure of the type filler - barrier layer - binder [5]. The rate of formation of the barrier layer in this case is limited by that of transport of the carbide-forming agent through the melt to the diamond surface. A drawback of this method lies in the slow rate of the process, as well as in the presence of an impurity, the carbide-forming agent, in the binder metal, which degrades its thermal conductivity. 

**Figure 1:**
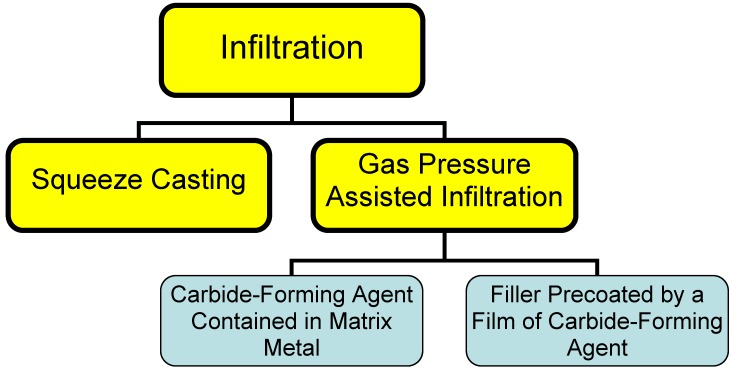
Different types of infiltration processes.

An alternative way of cutting the infiltration time consists in prior formation of a barrier layer on the diamond surface [12], with subsequent infiltration of the melted binder metal. A method was proposed [6] for deposition of a titanium coating on particulate nanodiamonds from NaCl melt at 1,000 °C, or from a NaCl + KCl + CaCl_2_ blend at 750 °C. Interaction of SiO_2_ with diamond in vacuum at 1,350 °C produces a uniform polycrystalline SiC coating 60 nm thick on diamond [13].

Infiltration can be used to advantage in fabricating large blocks of composites in an inert gas environment or under reduced pressure. The infiltration method is most expedient in production of large bulk heat sinks.

### 2.3. Spark plasma sintering

SPS is a fairly new approach to fabrication of composites from a variety of raw materials. Its main difference from the well-known methods employed in high pressure and temperature sintering lies in the possibility this approach offers of performing experiments in vacuum or an inert atmosphere and under pressure. A pulsed high-power current (500–1,000 kW) is passed through the sample to initiate spark discharge between particles of the powder to be sintered. This gives rise to local heating and efficient sintering of the composite, because what is actually being heated in the process is the sample material itself rather than the surrounding heater, as is the case with standard sintering techniques. The process could be conventionally characterized by two temperatures, more specifically, the average temperature of the material being sintered (it is usually not higher than 2,500 °C) and the local temperature at the discharge spot, which is substantially higher. The sintering process takes up, on the average, a few tens of minutes, whereas local heating lasts not longer than one millisecond.

This technique can be employed to advantage to sinter a broad variety of materials including high-strength metals (W, Co), ceramics (TiN, Al_2_O_3_), ZrO_2_- and Si_3_N_4_-based composites, diamond-metal composites, *etc*.

## 3. Models for Calculation of the Thermal Conductivity of Composites

Among the models employed to calculate the thermal conductivity *λ_c_* of composites of the filler-binder type obtained, as a rule, by infiltration the most popular is that of Maxwell [14]. This model assumes that the filler particles have spherical shape, and that the volume fraction of particles in the binder is not large (dilute medium). This model is applied, however, to composites with a large content of filler in the binder as well. Besides, the Maxwell model disregards the thermal resistance generated at the filler-binder boundary and the non-spheroidicity of the filler particles. This is why for such composites it yields overestimated values of thermal conductivity.

The Maxwell model for the thermal conductivity of diamond composites can be written in the following form:

(1) $$\lambda_{c}=\lambda_{m}\cdot \left[\frac{2\nu_{d}\left(\frac{\lambda_{d}}{\lambda_{m}}-1\right)+\left(\frac{\lambda_{d}}{\lambda_{m}}+2\right)}{\nu_{d}\left(1-\frac{\lambda_{m}}{\lambda_{d}}\right)+\left(\frac{\lambda_{d}}{\lambda_{m}}+2\right)}\right]$$

where *λ_m_* is thermal conductivity of the continuous matrix (for instance, copper), and *λ_d_* is thermal conductivity of a uniformly distributed filler (for example, diamond), *V_d_* is the volume fraction of spherical (diamond) particles.

It was shown, for example [15] that composites fabricated at a temperature of 1,473 K and a pressure of 4.5 GPa, in vials evacuated down to a pressure of 6.0 × 10^2^ Pa, from synthetic diamond grains of different size blended with copper, may have thermal conductivity both lower and higher than that of copper, depending on the volume fraction of diamond in copper and crystal size. The authors ascribe this to the copper-diamond boundary having a finite (2.97 × 10^7^ W/(m^2^·K)) thermal conductance. 

It is known that the contact thermal resistance at the boundary separating two phases in a composite originates from poor adhesion and a difference between the linear thermal expansion coefficients of the materials in contact [16,17,18]. This thermal resistance is called also the Kapitsa resistance, because it was Kapitsa who discovered in 1941 a break in the continuous temperature course at the metal-liquid interface [19]. One of the pioneering works dealing with a study of the thermal resistance forming at the diamond-metal boundary was published in 1977 [3]. A volume fraction of 0.64 is reportedly the maximum fraction to which a volume can be filled with randomly packed spheres.

It was shown [20] that for spheres stacked to form a hexagonal layered ordered structure the volume fraction is 0.74, and for ellipsoid-shaped spheres, it is 0.735 [21,22].

It thus appears obvious that one of the ways to improving the thermal conductivity of a composite lies in increasing the volume fraction of the filler in the heat-conducting matrix. A method to determine the filler volume fraction in a composite was proposed by Liang *et al.* [23].

There are other models by which one can calculate the thermal conductivity of heterogeneous composites as well. One could cite in this connection the model of Bruggeman proposed in 1935 [24]:

(2) $$1-\nu_{d}=\frac{\lambda_{d}-\lambda_{c}}{\lambda_{d}-\lambda_{m}}{\left(\frac{\lambda_{m}}{\lambda_{c}}\right)}^{1/3}$$

The Bruggeman model was refined to a version [25] in which, in the particular example of aluminum nitride added to polyimide, one took into account the effect of the filler particle shape and thermal resistance of the boundaries on thermal conductivity of the composite:

(3) $${\left(1-\nu_{d}\right)}^{n}=\left(\frac{\lambda_{m}}{\lambda_{c}}\right){\left(\frac{\lambda_{c}-\lambda_{d}(1-\beta )}{\lambda_{m}-\lambda_{d}(1-\beta )}\right)}^{n}$$

where *β* = *R_K_/R*; *R* is the radius of filler particles, *R_K_* is the Kapitsa radius, *R_K_* = *R_b_*·*λ_m_*, and *R_b_* is thermal resistance of the boundary. 

Hasselman and Johnson [26] modified the Maxwell equation to allow for the thermal resistance at the filler-binder boundary

(4) $$\lambda_{c}=\lambda_{m}\frac{2\left(\frac{\lambda_{d}}{\lambda_{m}}-1-\frac{\lambda_{d}}{r\cdot G}\right)V_{d}+\frac{\lambda_{d}}{\lambda_{m}}+2+2\frac{\lambda_{d}}{r\cdot G}}{\left(1-\frac{\lambda_{d}}{\lambda_{m}}+\frac{\lambda_{d}}{r\cdot G}\right)V_{d}+\frac{\lambda_{d}}{\lambda_{m}}+2+2\frac{\lambda_{d}}{r\cdot G}}$$

where *r* is the radius of spherical filler particles, and *G* is the boundary thermal conductance.

To take into account particle shape, the Maxwell model thermal conductivity of a composite was further modified by Hamilton and Crosser [27]:

(5) $$\lambda_{c}=\lambda_{m}\left(\frac{\lambda_{d}+(n-1)\lambda_{m}+(n-1)\nu_{d}(\lambda_{d}-\lambda_{m})}{\lambda_{d}+(n-1)\lambda_{m}-\nu_{d}(\lambda_{2}-\lambda_{1})}\right)$$

where *n* is a factor allowing for particle sphericity, *n* = 1/Ψ, Ψ takes into account sphericity; Ψ = 1 for spherical particles.

The thermal conductivity model was further upgraded in a study [28] of the specific features of diamond packing in a composite with metal binders Al, Cu, Ag. The authors took here into account the effect of such parameters as the volume fraction of diamonds, size of the filler and particularly crystal habitus on the thermal conductivity of a composite and thermal resistance at the diamond-binder interface.

Significantly, different crystallographic faces of diamond differ in wettability. It was experimentally demonstrated [29,30] that aluminum wets well the {001} square faces of diamond while not adhering to the {111} hexagonal faces. It is suggested that thermal conductance at the diamond-aluminum boundary depends typically on the face, with *G_001_* = 1 × 10^8^ W/(m^2^·K) and *G_111_* = 1 × 10^7^ W/(m^2^·K) [28].

These results are corroborated by Chu *et al.* [31], but this study revealed a possibility of formation of diffusion bonds between aluminum and the {111} faces of diamond in Al(Si)-based composites fabricated by gas pressure infiltration.

A model developed to quantify thermal conductance of boundaries in a polycrystalline composite [32] addresses the dependence of thermal conductivity of polycrystalline yttria-stabilized zirconia on particle size within a broad temperature interval ranging from 6 K to 480 K:

(6) $$\lambda_{c}=\frac{\lambda_{d}}{1+\frac{\lambda_{d}R_{k}}{d_{d}}}$$

where *R_k_* = 1/*G*, *G* is the Kapitsa conductance.

Practically the same equation was obtained by Nan *et al.* [33] in terms of an effective medium approach combined with the essential concept of Kapitsa thermal contact resistance, Eq. (6) in the abovementioned paper, which differs from the above expression only in the factor two included in the second term of the denominator.

Yang *et al.* [32] believe that this difference should be assigned to not having taken into account that each grain boundary is common for two grains.

This model was used by Angadi *et al.* [34] to calculate thermal conductivity of a ultrananocrystalline diamond film. The experimentally determined thermal conductivity of the film was found to be *k* = 15 W/(m·K), the thermal conductivity of a diamond core 3–5 nm in size was assumed equal to *k* = 2,200 W/(m·K), and for that of the boundaries separating nanodiamonds one obtained 3 GW/(m^2^·K). The value of the boundary thermal conductance thus found exceeds about tenfold that of any other substances.

This model was further refined [35] by invoking mesoscale computer simulation to study the effective thermal conductivity of two-dimensional polycrystalline model microstructures containing finely dispersed stationary voids.

A model developed for calculation of the elastic stresses generated in a filler-binder-type sample by the finite element method for a particular case of Al-SiC was described by Prabu *et al.* [36].

To find thermal resistance at the boundary between two phases, one usually invokes the acoustic mismatch model (AMM) or diffuse mismatch model (DMM) [14,37]. The main parameters in AMM are the boundary transparency *τ_AMM_* for acoustic phonons and the phonon densities of states:

(7) $$\tau_{AMM}=\frac{4z_{1}z_{2}\cos(\theta_{1})\cos(\theta_{2})}{{\left(z_{1}\cos(\theta_{1})+z_{2}\cos(\theta_{2})\right)}^{2}}$$

where *z*_1_ = *ρ*_1_*υ*_1_, *z*_2_ = *ρ*_2_*υ*_2_ are acoustic impedances, *ρ* is the density, *υ* is the velocity, and *θ* is the angle between the normal and the phonon propagation direction.

This model was upgraded by taking into account the binding forces coupling atoms on two different surfaces [38], which could be of significance for carbon nanosized objects which can be coupled both by covalent and van der Waals forces. 

We may recall that the mean free path for phonons in most substances does not exceed 1 nm even in the low-temperature domain. One can therefore safely assume that at a boundary phonons should scatter by the diffusion mechanism, in other words, that the model appropriate here is DMM.

DMM was further extended in [39,40]. Duda *et al.* [39] included high elastic anisotropy of one of the two materials in contact and found 30 W/(m^2^·K) for thermal conductance of the Al-graphite boundary (c-axis).

Reddy *et al.* [41] allowed for the spectrum of phonons being nonlinear. Thermal conductances of the Al-Si, Al-Ge, Cu-Si, and Cu-Ge interfaces was determined for the temperature range extending from 0 K to 800 K. The thermal conductances of these interfaces were found to vary from 0.1 GW/(m^2^·K) to 0.3 GW/(m^2^·K) at 300 K. Hopkins [40] proposed a new model which includes multiple phonon processes occurring in transmission of the interface between two solids.

## 4. Composites Fabricated by High-Pressure, High-Temperature Diamond Sintering

Research conducted presently in the field of high-pressure diamond-based materials is focused primarily on development of composites which would demonstrate high hardness and/or thermal conductivity. Consider in some detail the main features and characteristics of sintering of microcrystalline diamond and microcrystalline diamond with various binders, with particular emphasis being placed on sintering of detonation nanodiamond.

**Figure 2:**
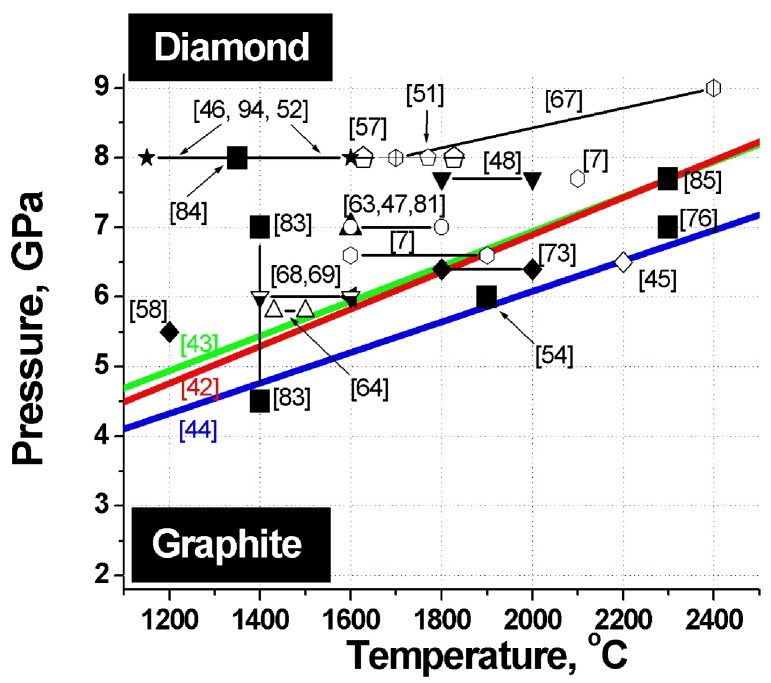
Phase diagram of carbon. Heavy lines - graphite-diamond phase equilibrium lines. Large filled squares - sintering parameters of detonation nanodiamond composites. The other symbols refer to the pressures and temperatures at which samples of microcrystalline diamond differing in size and habitus, both from pure diamond and from its mixtures with titanium, silicon, copper and other binders were obtained. The corresponding references are specified in parentheses.

To identify the optimum conditions for fabrication of diamond-based composites with the best possible properties, let us analyze the phase diagram of carbon, which features superposed diamond-graphite phase equilibrium lines obtained by different researchers, and sintering parameters of a variety of diamonds (Figure 2). The heavy lines specify diamond-graphite phase equilibrium conditions found by Cannon 1962 [42], Kennedy 1976 [43] and Bundy 1996 [44].

Inspection of this diagram suggests a conclusion that the best samples are obtained in conditions close to those of the diamond-graphite phase transition, *i.e.,* where diamond crystals undergo surface graphitization. These facts are taken from appropriate papers indicated on Figure 2.

### 4.1. Sintering of microcrystalline diamond

A pioneering work on the thermal conductivity of sintered diamond appeared in 1974 [45]. It addressed the possibility of developing diamond-based heat sinks for semiconductor devices. It was demonstrated that samples exhibiting the highest thermal conductivity are obtained at a pressure of 6.5 GPa and a temperature of 2,200 °C.

Shulzhenko *et al.* [46] prepared polycrystalline synthetic diamond containing up to 1.5% impurities and inclusions, 10–14 µm in size, which were sintered in the region of diamond stability at a pressure of 8 GPa and at power levels from 2.4 kW to 4.2 kW. These samples were used to study the dependence of the strength (twisting), hardness and wear resistance, as well as of the density, thermal conductivity, and electrical resistance on sintering power (temperature). It was shown that the hardness of polycrystals increases from ~20 GPa to 75 GPa and depends close to linearly on sintering power within this power interval. The thermal conductivity increases from 60 W/(m·K) to 190 W/(m·K), and the electrical resistance drops sharply from over 10^6^ Ohms at 2.6 kW down to 10^3^ Ohms at 3 kW, which should be ascribed to graphite forming on open surfaces of diamond grains. The size of diamonds at a section was 5–10 µm.

The behavior of the electrical conductivity of the samples fabricated by sintering of ASM 40/28 synthetic diamond powder at 7 GPa in the diamond stability region with time, and its dependence on sintering temperature were reported by Sokolina *et al.* [47]. It was shown that at sintering temperatures above 1,200 °C the electrical resistivity does not exceed 10^3^ Ohms·m and drops to 10 Ohms·m at 1,600 °C. The electrical resistivity becomes practically independent of sintering time for times over 10 s, which should possibly be assigned to the temperature in the high pressure chamber reaching a steady-state level.

“Pure” particulate diamond, ~4 µm in size, can be synthesized by sintering at 7.7 GPa, 2,000 °C, in 1 hour without formation of concomitant graphite as well [48]. Moreover, it is believed [49,50] that graphitization of microcrystalline diamond, 30–40 µm in size, at a pressure of 2 GPa occurs at temperatures above 1,200 K because of the powder containing oxygen and residual water vapor. Indeed, one succeeded in fabricating at a pressure of 2 GPa samples which contained practically no graphite at all even at a temperature of 1,760 K when sintered for 20 min. Graphitization of diamond starts from the surface, and graphite crystals grow in the direction perpendicular to the surface and along it.

The experiments of Voronov *et al.* [51] demonstrated that sintering of natural diamond crystals of metamorphic rocks (yellowish-green coloring, high nitrogen concentration, 5.9 × 10^18^ at/cm^3^, in the disperse phase and 2.1 × 10^20^ at/cm^3^ in the exchange-coupled form) performed in toroid-type equipment at a pressure of 8 GPa and temperature of 1,800 °C for 30 s occurs by diffusion mechanism. It was shown that the density of compacted samples does not depend on time and sintering temperature and reaches 3.3 ± 0.1 g/cm^3^ (measured picnometrically) and 3.05 ± 0.1 g/cm^3^ (apparent density). The samples were boiled in hydrochloric acid for 25 h, after which their picnometric density increased to 3.5 ± 0.01 g/cm^3^, thus suggesting that non-diamond *sp^2^* carbon was totally removed, a conclusion corroborated by the electrical resistivity measured to be not less than 10^11^ Ohms·cm.

This implies that the composites have open porosity; said otherwise, there is no indication of plastic flow of diamond at the sintering parameters specified. The thermal conductivity of the composites produced by sintering 20-µm particulate diamond powder was at a level of 250 W/(m·K). Sintering of diamond powder involves two concomitant and competing processes, more specifically, recovery of diamond *sp^3^* bonds linking the grains and phase transformation of diamond into *sp^2^* carbon at the pore surface. While both processes become intensified with increasing temperature and heating time, they act in opposite directions on the thermal conductivity. The maximum compressive strength of cylindrically shaped, dia. 4 mm and 3 mm high, diamond samples with end faces ground properly was found to be 4.1–4.2 GPa, and it did not change after heating in an inert medium to 1,200 °C.

The porosity of the composites fabricated from crystals increases as their size decreases. Bochechka *et al.* [52] studied sintering of ASM 40/28, 28/20, 20/14, 5/3, 0.3/0 and 0.1/0 diamonds at 8 GPa and a temperature of 1,550 °C. It was shown that the sample density reaches a maximum of ~3.4 g/cm^3^ after 10–12 s of sintering, after which it no longer depends on sintering time. The density of ASM 0.3/0 diamond samples which had been preliminarily degassed in vacuum at 500 °C did not exceed 3.1 g/cm^3^.

The thermal conductivity of composites prepared from natural particulate diamond 10–14 µm in size was as high as 500 W/(m·K); the samples that featured maximum values of thermal conductivity were fabricated at the pressures and temperatures close to the diamond-graphite phase equilibrium boundary [53,54].

As in composites featuring a high thermal conductivity, a significant role in abrasive composites is played by the quality of crystal fixation in the matrix and the processes occurring at the boundary. Chemical binding to the matrix through formation of carbides appears to be the most efficient method to fix the diamond in the matrix. As a rule, carbides form on the surface of diamonds through chemical reaction of carbon in the form of graphite with the matrix component. Intense graphitization of diamond is known to start at temperatures above 1,000 °C.

It is known also that the rate of graphitization at different graphite planes is different [55,56] and depends on diamond grain size. It was shown [57] that (i) the rate of transformation of (111) diamond planes to graphite-like sheets is higher than that of the other planes, (ii) the edges of exfoliated graphite-like sheets merge with the upper untransformed diamond planes, and (iii) the distance between the inner graphite-like sheet and the upper untransformed diamond layer does not exceed 0.35 nm, which implies coupling between the diamond and graphite layers.

Graphitization of the {100} and {111} faces of diamond crystals at pressures of 0.1 and 2 GPa and different temperatures was studied by Raman spectroscopy, X-ray single-crystal diffractometry and scanning electron microscopy [55]. Different primary mechanisms of graphitization are discussed, among them (i) normal graphite layer growth through detachment of single atoms from the {100} and {111} diamond surfaces and (ii) lateral growth of graphite on the {111} surface through detachment of groups of atoms followed by their rearrangement into planar graphitic structures. Growth of oriented graphite crystallites was observed to occur only on the {111} diamond faces and at *p* = 5.2 GPa. Internal graphitization of synthetic diamonds was observed and explained in terms of catalytic effects on metal inclusions.

The use of high pressures paves the way to reaching higher temperatures which are needed to melt the binder materials and form their carbides. At the same time, at pressures above 2 GPa microcrystals which measured originally 30–40 µm break up into fragments down to 50–80 nm in size [49]. The dislocation density grows as sharply. Decrease in size to 100 nm is observed also for diamonds compacted at up to 8 GPa at 1,470 K [58], as well as for ASM 7/5 diamonds whose size after compression decreased to considerably less than 0.5 µm [59]. The decrease in size and growth of dislocation density in diamond crystals degrades naturally the thermal conductivity of the composites obtained.

### 4.2. Sintering of microcrystalline diamond with the binder metal

For the binder metal one most frequently chooses refractory metals, such as titanium, cobalt, tungsten, *etc*, which makes it possible to use composites at a high (~800 °C) temperature. The thermal conductivities of Ti, Cr, Mo, W and their carbides lie in the range of 7–170 W/(m·K), which makes it hardly reasonable to expect composites with these metals to have high thermal conductivities. Refractory metals are known to form carbides. The temperatures at which composites are fabricated are, as a rule, higher than the melting point of the metal or of its carbide. The Table 2 below lists melting points of the eutectics of some metals with carbon and the carbon concentration in the solid and melted metals [60]. 

**Table 2:** Solubility of carbon in metals [60].

Element	Melting temperature of metal-carbon eutectic, °C	Carbon concentration in solid metal (at %)	Carbon concentration in melted metal (at %)
Ti	1648	3.1	1.96
V	1650	4.3	15.0
Cr	1534	0.3	13.9
Mn	1243	11	11.1
Fe	1153	9.1	16.9
Co	1321	4.5	11.9
Ni	1327	2.7	6.93

The melting point of titanium carbide, TiC, is 3,067 °C, and that of tungsten carbide, 2,648 °C [4]. One has to maintain, besides a high temperature, a high pressure as well in order to support thermodynamic stability of the diamond. Recall that the melting temperature of a metal increases with increasing pressure. For most catalyst metals employed in industry for diamond fabrication (Fe, Ni, Co, Cu) the rate of variation of the melting point is approximately 35 °C/GPa within the pressure interval from 0 GPa to ~6 GPa (Table 2) [61].

It was experimentally demonstrated that metals penetrate into diamond at 3 GPa and 1,800–2,300 K in 10 s; no traces of metal penetration into graphite under the same conditions were, however, revealed. At 9 GPa and 1,800–2,300 K, metals penetrate into bulk graphite in 5–10 s as intensely as they do into diamond powder [62]. Liquid iron-group metals penetrate into diamond powder at any pressure, but not into graphite in 10 s of observation, unless the latter underwent phase transition to diamond. Diamond subjected to high pressure reveals in the region of its thermodynamic stability formation between powder grains of non-diamond carbon of turbostratic structure.

Of considerable interest are carbonado-type composites made up of diamond and Group VIII metals which are capable of dissolving it. They are usually fabricated by placing a metal wire seed at the center of a diamond or graphite sample, with its subsequent high-pressure and high-temperature treatment. A study was made of the shape of diamonds in polycrystals obtained at 7 GPa, 1,600 °C under 5-min sintering [63]. Melting metal is squeezed into diamond producing characteristic extended structures aligned with the direction of metal flow. On the other hand, if cobalt present in amounts of under 5 vol % is distributed uniformly in the original diamond-metal mixture, then 1-h sintering at 7.7 GPa and 2,000 °C will produce composites with a high electrical resistivity of 1.3 × 10^8^ Ohms·cm (such amounts of cobalt do not form continuous conducting channels) and a hardness of 100–150 GPa, which is in excess of that of single-crystal diamond of type Ib (56–108 GPa) and IIb (75–115 GPa) [48]. Homogeneous polycrystalline composites of 0.5-µm diamond and cobalt (5–10 vol % of diamond mass) can be obtained at a pressure of 5.8 GPa and temperature of 1,430–1,480 °C only by adding to the mixture a small amount of cubic boron nitride, which prevents growth of large diamond crystals [64]. One succeeded in fabricating at 6.5 GPa and 1,600–1,900 °C heat-resistant composites from synthetic diamond (2–4 µm in size) to which TiC_0.6_ or TiC was added [7]. It is reported that homogeneous structures with a hardness of 45 GPa (by Vickers) could be fabricated at 6.5 GPa only at temperatures above 1,800 °C. These composites revealed noticeable degradation after heating at 1,500 °C for 30 min in vacuum.

As a rule, the mechanical properties of diamond-metal composites with carbide-forming or refractory metals used are superior to those of composites fabricated from pure diamond. One should naturally keep in mind that a strong difference between the thermal expansion coefficients of diamond (1.18 × 10^–6^ K^−1^) and a metal, for instance, of cobalt (12 × 10^–6^ K^−1^) is capable of generating intense internal stresses which can initiate formation of microcracks in the diamond [65]. It is pointed out [66] that in order to wet diamond to obtain a high-quality sintered sample, cobalt should be added in an amount of 16–20 vol %. Moreover, interaction of diamond with cobalt under pressure of 5.8 GPa passes through an intermediate stage. In the first step, the diamond surface is graphitized at about 1,300 °C (the diamond-cobalt eutectic temperature is 1,336 °C). As the temperature is increased to 1,400 °C, the graphite formed on the diamond surface dissolves in cobalt to convert back to diamond. Diamonds ranging in size from 0–1 µm to 5–12 µm were used in the sintering. The composites fabricated from the 5–12-µm particulate diamond exhibited lower microhardness (2,500 kg/cm^2^) than those from the 0–1 µm lot. The authors are inclined to assign this to graphitization of 0–1 µm particles occurring at lower temperatures than that of particles 5–12 µm in size.

Sintering of 3–5-µm diamond with WO_3_ at pressures of 8–9 GPa and temperatures of 1,700–2,400 °C performed for 60 s is reported by Ekimov *et al.* [67]. The sample sintered under these conditions revealed the presence of graphite, as well as of tungsten compounds WO_3_, W_3_O_8_, and W_2_O.

It was demonstrated [8] that thermal conductivity of composites fabricated at a pressure of 8 GPa and temperatures of 1,300–2,100 K from particulate diamond of different sizes and copper increases from 200 W/(m·K) to 800 W/(m·K) with increasing diamond filler size from 10 µm to 500 µm.

### 4.3. Sintering of microcrystalline diamond with a nonmetallic binder

There is a variety of composites of diamond with a nonmetallic binder. One can use as binder silicon nitride [68], silicon carbide [69], silicon and aluminum nitride [70]. The properties of polycrystals obtained in the diamond-silicon-carbide system start to degrade already at a temperature of 1,200 °C, even at low silicon concentrations. Ko *et al.* [69] sintered diamond powder 0.1–1 µm in size with SiC (0.1–1 µm) and aluminum (3 µm). SiC was added to diamond in amounts of 10–50 wt %. Sintering was run at 6 GPa and 1,400–1,600 °C for 1 h. X-ray diffraction patterns revealed weak peaks identified with graphite, but their intensity fell off with increasing SiC concentration in the diamond. As the temperature was increased from 1,400 °C to 1,600 °C, and the diamond concentration, from 50% to 90%, the Knoop hardness of the composites increased from 25 GPa to 35 GPa. Sintering did not affect grain size, and no indications of SiC melting were observed.

Noma *et al.* [70] report on a possibility of fabricating diamond-silicon carbide composites at pressures of both below 3 GPa (region of graphite stability) and above 5 GPa. It was shown that SiC forms on the surface of diamond and is accompanied by diffusion of silicon and carbon through the SiC layer thus formed. Significantly, the activation energy of silicon carbide formation was found to depend on the phase, graphite (pressures below 3 GPa) or diamond (pressures above 5 GPa), from which carbon diffuses through SiC. The activation energy of SiC formation was determined to be 410 ± 182 kJ/mol at 2 GPa and 1,765–1,975 K, and 264 ± 51 kJ/mol at 9 GPa and temperatures of up to 2,575 K.

A Raman spectroscopy study [71] was made of the dependence of the stresses in diamond crystals appearing in the interaction of diamond with silicon carbide on sintering temperature, pressure and crystal size. The samples were fabricated at pressures of up to 10 GPa and temperatures of up to 2,273 K. It was demonstrated that the stresses are initiated by the differences between the thermal expansion coefficients and bulk moduli of diamond and silicon carbide.

At a pressure of 8 GPa, the activation energy of carbon interaction with silicon atoms separated by a SiC film (in the region of thermodynamic stability of diamond) is 264 kJ/mol [72,73]. The process of SiC formation is limited by the rates with which carbon and silicon atoms diffuse through the SiC film. At 2 GPa, SiC can form in the region of graphite thermodynamic stability both directly from diamond and in two stages. In the first stage, diamond converts to graphite, which subsequently reacts with silicon. As a result, the activation energy for SiC formation increases to 410 kJ/mol. For nanosized diamond, in the region of its thermodynamic stability the activation energy of SiC formation is 170 kJ/mol, and silicon starts to react with carbon below the silicon melting temperature [74]. The authors assign this to nanodiamond having a larger number of strained grain-boundary bonds. 

A study was performed of the samples obtained by silicon infiltration at 8 GPa and 2,170 K into particulate diamond of different sizes [75]. The samples were found to have a distinct nanocrystalline structure; indeed, the volume-weighted mean crystallite size is 41–106 nm for the diamond phase and 17–37 nm for the SiC phase. The decrease of diamond crystal size leads to increased dislocation density in the diamond phase, lowers average crystallite size in both phases, degrades composite hardness and improves fracture toughness. 

A study was reported of the effect of particle size of dynamically synthesized nanodiamond powders on silicon infiltration and SiC phase formation [76,77]. It was established that silicon does not penetrate into the pores of nanodiamond powder if the original particle size is smaller than 0.5–1.0 µm. The critical pore size for infiltration is 100–200 nm. A study of the microstructure of the samples revealed the presence of a nanometer- and submicron-scale SiC phase. The rate of propagation of a longitudinal sound wave in such composites reached 14.57 km/s, and that of the transverse one, 10.07 km/s. The measurements of the sound velocity permitted determination of the elastic moduli.

Ekimov *et al.* [8] demonstrated that Ti and Si present in the diamond composites fabricated at a pressure of 2 GPa are not just a filler but form a binder as well. The thermal conductivity of such composites depends only weakly on diamond grain size and may be as high as 600 W/(m·K) for composites with a SiC-Si matrix.

### 4.4. Sintering of detonation nanodiamond (DND)

A model describing the structure of a diamond nanocluster was developed using X-ray diffraction and small-angle scattering data [78]. DND was isolated from detonation soot by etching the latter in 50% and 70% nitric acid in an autoclave at temperatures varied from 180 °C to 260 °C. Particulate DND was shown to consist of a diamond core, an onion-like carbon sheet, and a layer of graphite nanoplatelets, atop which graphite particles and metal oxide inclusions are dispersed. Oxidation leaves the nanodiamond core and a sheet of onion-like carbon intact, although this sheet can be readily removed, for example, by ozone etching.

DND is typically 4.5–10 nm in size, depending on the actual conditions of its formation in the shock wave. The fraction of surface atoms of nanodiamond particles ranging in size from 2 nm to 10 nm varies from 63% to 15%, which corresponds to the number of internal carbon sheets from 4 to 18 [79].

The number of publications dealing with high-pressure sintering of detonation nanodiamond is presently scarce [53,54,80,81,82,83,84,85]. The pioneering papers appeared in 2004. It appears presently universally accepted that microcrystalline diamond differs radically from detonation nanodiamond in both thermodynamic and kinetics properties.

Compare first detonation nanodiamond with microcrystalline diamond from the viewpoint of their thermodynamic stability, after which we shall turn to the specific features of high-pressure detonation nanodiamond sintering.

### 4.5. Comparison of the thermodynamic stability of nanodiamond and microcrystalline diamond

DND graphitization in vacuum starts already at 900 °C, substantially below the graphitization temperature of bulk single-crystal diamond (>1,900 °C) [86]. An analysis of Raman spectra and X-ray diffraction patterns of DND whose sintering was performed at different temperatures suggests a conclusion that at temperatures above 720 K *sp^2^* fragments present in the original DND undergo ordering [86]. Annealing of nanodiamond in air above 800 °C makes it possible to reduce particle size from ~50 Å to ~25 Å, whereas bulk diamond starts to graphitize only at a temperature of ~1,500 °C [87].

**Figure 3:**
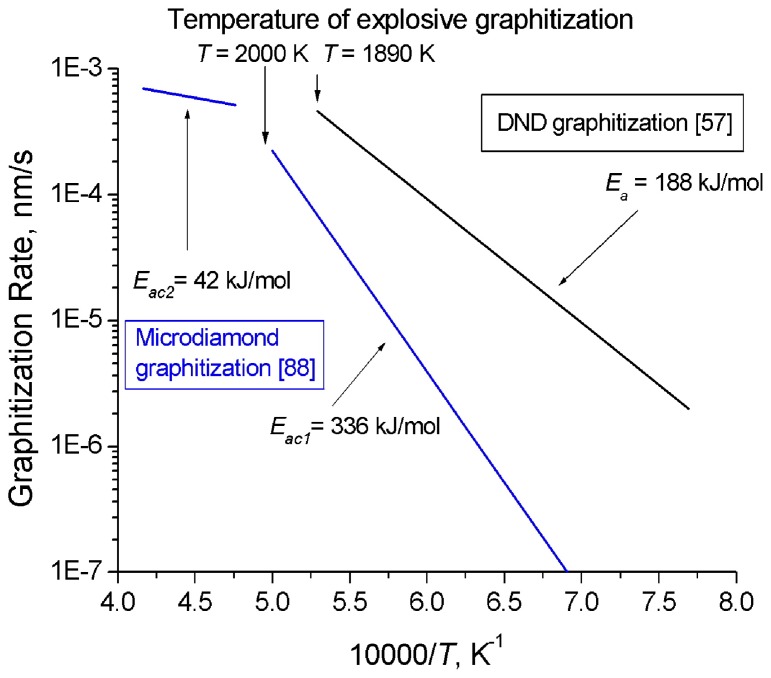
Behavior of microcrystalline diamond [88] and DND [57] graphitization rate with temperature.

Andreev [88] invoked the method of model potential of atomic interactions to develop a theoretical description of the mechanism and kinetics of diamond graphitization occurring with two strongly different activation energies and accompanied by an explosive destruction of the graphitizing diamond crystals. As demonstrated experimentally, the rate of diamond graphitization varies in the course of diamond powder annealing at temperatures of 1,900–2,200 K, with two linear sections in the ln(*C_G_*) = *f*(*T*^-1^) seen clearly (*C_G_* is the graphite content in the sample after 30 min of isochoric annealing), Figure 3. The activation energy *E_ac1_* = 336 ± 21 kJ/mol (for T < 2,000 K) relates to the diffusion mechanism, and *E_ac2_* = 42 ± 8 kJ/mol (for T > 2000 K), to the explosive mechanism. Thus, graphitization proceeding with the low activation energy E*_ac_*_2_ starts at higher temperatures than the process with the higher one, *E_ac1_*. The reaction rate constants calculated from the Arrhenius equation (8) for the rate of formation of the new phase:

(8) $$d\alpha /dt=A_{0}\exp(-\frac{E_{ac}}{RT})$$

and the experimentally determined activation energies are *A_01_* = 8 × 10^8^ %/min = 1.33 × 10^5^ s^-1^ and *A_02_* = 34 %/min = 5.7 × 10^–3^ s^-1^, *i.e.*, they differ by approximately seven orders of magnitude. This equation does not provide an answer to the question of why a lower activation energy initiates the process of spontaneous graphitization only at a very high temperature. The explanation for this phenomenon should be looked for in the atomic mechanism of spontaneus graphitization which involves two barriers, the energy barrier of bond dissociation *D_0_* and a force barrier of bond “yield strength” *F_max_*, which initiates the spontaneous graphitization mechanism. The second barrier can be identified with a certain threshold energy and temperature.

Kuznetsov *et al.* [57,89] also considered the process of nanodiamond graphitization and proposed a model for formation of the onion-like carbon. The activation energy of DND graphitization *E* = 188 ± 21 kJ/mol, *A_0_* = 74 ± 5 nm/s at a temperature of less than 1,910 K (Figure 3). These experiments suggest convincingly that bulk diamond is kinetically more stable than nanodiamond.

By contrast, Zhao *et al.* [90] showed the energy of DND transformation to nanographite to depend on the size of DND particles. The authors assign this relation to the existence of a certain additional pressure acting on nanoparticles; this pressure is believed to originate from surface tension and bending, which decrease with increasing nanoparticle size. As the particle size and temperature decrease, the stability of diamond relative to graphite increases [91]. Graphitization of diamond Ia and of nanodiamond obtained from C_60_ at a pressure of 20 GPa and a temperature of 2,000 °C was studied in Ar + 2% H_2_ inert atmosphere experimentally [92]. It was found that thermal expansion of diamond Ia starts at 1100 K and becomes very fast at 1,500 K, whereas nanodiamond reveals signs of changing only at 1,900 K. 

The phase diagram of carbon plotted *vs*. particle size suggests that the temperature of the diamond-graphite phase transition increases with decreasing diamond size [56,93]. These experiments provide supportive evidence for nanodiamond being kinetically more stable than bulk diamond. Data obtained on nanodiamond sintering under pressure (see Figure 2) show persuasively that at pressures of 3–9 GPa nanodiamond is more stable than microcrystalline diamond.

### 4.6. High-pressure sintering of nanodiamond. Properties of the composites obtained

The interest in high-pressure sintering of detonation diamond is prompted not only by expectations of obtaining a material with properties typically seen in composites made from bulk diamond but also by the possibility of observing in it such phenomena as an increase in particle size, variation of the thermodynamic stability of diamond with particle size, and effect of modification of nanodiamond surface on the properties and parameters of fabrication of this composite.

Senyut *et al.* [81] study the dependence of the microhardness of composites made from DND and DND modified by cobalt and titanium on sintering temperature at a pressure of 7 GPa. We note that at 1,300 °C the density of DND samples is 2.73 g/cm^3^, and microhardness—20 GPa, while at 1,600–1,800 °C they are 2.85–2.90 g/cm^3^ and 30–35 GPa. Significantly, the sample has 2–5 µm micropores totaling up to 5% over the section area, and 1–18 µm micropores over 15% of section area. A sample prepared at 2300 °C exhibits a branched network of microcracks, density of 2.7 g/cm^3^, and microhardness of 14–16 GPa, and it was found to contain graphite. Products of DND sintering in vacuum were demonstrated to have scales 100–750 µm in size with microhardness of 10–50 GPa, and particles measuring 700–750 µm with a characteristic diamond habitus and microhardness at a level of 100 GPa.

By modifying DND with boron, titanium, and silicon, one can bind oxygen which, while giving rise to diamond graphitization, brings about formation of carbides as well [80]. 

Shul’zhenko *et al.* [94] investigated the dependence of the strength and hardness of polycrystalline DND on sintering conditions, 1,150–1,600 °C at 8 GPa, and prior surface modification of DND particles. Directly before sintering, the samples were subjected to heat treatment in vacuum at T = 500 °C to remove the gases adsorbed on DND surface. It was found that with the amount of gas on the DND surface decreasing, the density of the composites increases up to 2.86 g/cm^3^, and their strength reaches as high as 28 GPa. Removal of chemisorbed carbonyl groups and the noticeable decrease in the number of hydroxyl groups (as revealed by IR spectroscopy) practically exclude completely graphitization of the powder in sintering at 1,550 °C and a pressure of 8 GPa. The hardness of the samples was demonstrated to scale linearly with their density.

DND sintering at a pressure of 4.5–7 GPa and temperature of 1400 °C for 3 min was studied by Yushin *et al.* [83]. After the sintering, the sample was crushed down to particles 1–6 µm in size, treated for 30 min in a solution of 50% sulfuric acid and 50% chromic anhydride, and washed subsequently in distilled water. This very hard chemical treatment of the sintered sample culminated in conclusions that should be qualified, in our opinion, as nothing else but inadequate. Indeed, the maximum pore size is stated by the authors to be 5 nm, and it is conjectured that the content of onion-like carbon and amorphous carbon in the sample is less than that in the original DND. The maximum size of a diamond particle did not exceed 27 nm.

Davydov *et al.* [84] report on DND sintering at 8 GPa within the temperature interval of 873–1,673 K for 60 s in a toroid-type chamber. It is believed that sintering brings about increase of DND size from 4.5 nm to ~18 nm.

The increase of average DND particle size under sintering could be ascribed to small particles becoming sintered at lower temperatures than larger ones do [95]. This, however, is in clear contrast to XRD data referred to above.

Diamond single crystals with decahedral and icosahedral faceting and dimensions of 50–80 nm were obtained by high temperature (2,300 °C) and high pressure (7.7 GPa) treatment of detonation nanodiamonds [85]. It is concluded that the growth of diamonds is related to the oriented association of initial nanodiamond nanoparticles, the driving force of which is the tendency of the system to reach the state of minimum surface energy.

Sintering of DND particles 3–5 nm in size with SiC (28 vol %) at 8 GPa and 1,700–1,800 °C for 1 min [77] yielded samples which did not contain the silicon and graphite phases, with a hardness of 100 GPa, density of 3.4 g/cm3 and velocities of propagation of the longitudinal and transverse ultrasonic waves of 14.57 and 10.07 km/s, respectively. For the composite obtained from DND in similar conditions, the hardness was 25 GPa, density of 3.1 g/cm^3^, and ultrasound velocities of 12.79 km/s (longitudinal) and 8.73 km/s (transverse). At temperatures below 1,600–1,700 °C, silicon envelops DND uniformly, and reacts with increasing temperature with carbon to produce submicron-sized SiC crystals.

Thus, sintering of pure DND does not provide a way to reaching a noticeable increase of diamond particles in size. The size of particles does not usually exceed 30 nm, which immediately implies a large number of grain boundaries. Besides, the density of the composites thus obtained is not over 3 g/cm^3^, which evidences a high porosity and presence of the *sp^2^* phase in the samples. It is apparently these two factors that account for the low thermal conductivity of sintered DND.

Kidalov *et al.* [54] measured the thermal conductivity of DND sintered at a high pressure (6.5 GPa) and found it to range from 5 W/(m·K) to 50 W/(m·K), depending on the actual sintering temperature. Similar values of thermal conductivity are typical also for polycrystalline diamond films with crystal size of 3–5 nm; indeed, they are 12 W/(m·K) [34], 13 W/(m·K) [96], and 4.5 to 13.7 W/(m·K) [97].

Assuming the thermal conductivity of a nanodiamond core to be 2,000 W/(m·K), the equation (6) for thermal conductivity of polycrystalline material yields 1–4 GW/(m^2^·K) for the thermal conductance of boundaries between nanodiamonds in a composite. Such figures are apparently typical of all nanosized objects [98].

### 4.7. Conclusions to the chapter

Sintering of microcrystalline diamond gives rise to surface graphitization of diamond crystals. Samples demonstrating the highest values of strength and thermal conductivity are obtained when diamond is sintered close to the diamond-graphite phase equilibrium boundary. Composites based on microcrystalline diamond with a metallic binder feature a high strength and are used to advantage in abrasive technology. Composites produced from microcrystalline diamond with silicon binding can find application as materials for heat sinks.

As for detonation nanodiamond, of most interest for research, both in the area of possible applications and from the standpoint of basic science, would be uncovering the mechanism of DND sintering which brings about an increase of their size, the mechanism of explosive DND graphitization, determination of the DND graphitization rate, a search for a possibility of DND sintering at lower pressures and temperatures, in particular, through modification of the developed DND surface, as well as for a way to fabrication of large microcrystalline diamonds (up to 600 µm) by DND sintering.

Of particular significance would be to analyze the possibility of chemical modification of DND surface. Such a modification could apparently affect markedly the properties of the samples obtained.

## 5. Composites Fabricated by Infiltration

The most impressive progress in fabricating composites with a high thermal conductivity by infiltrating copper into diamond was announced in Refs. [5,12,15,99]. The thermal conductivity of composites was reported to reach 700 W/(m·K) in [12,15,99], and 640 W/(m·K), by Schubert *et al.* [5].

Aluminum was studied as a candidate for use as matrix material [29,30,100]. The thermal conductivity of such composites reached as high as 670 W/(m·K) [29,30] and 580 W/(m·K) [100], which we believe to be an outstanding achievement for aluminum-based composites.

In order to make matrix metals based on copper, aluminum, *etc*. wet diamond, the carbide-forming metals B, Si, Ti, Cr, W are used. The need for formation of carbides on the surface of diamond is adequately illustrated by the data of Weber *et al.* [99] which demonstrate the dependence of the thermal conductivity and thermal expansion coefficient of the diamond-copper/chromium composite on the concentration of chromium in copper. The authors observed a transition of the diamond/matrix from weak to strong bonding. This gives rise to growth of the thermal conductivity (to over 600 W/(m·K)) and decrease of the thermal expansion coefficient (to less than 10 × 10^–6^ K^−1^) of the composites.

Thermal conductivities of the refractory metals and carbides of the elements specified above lie usually in the 7–170 W/(m·K) range; therefore, thickness of the coating affects markedly the total thermal conductivity of the composite. The thickness of the carbide coating at which thermal conductivity of the composites reaches a maximum is, as follows from electron microscopy measurements, 100–200 nm.

These elements form carbides in the following reactions:

C_Dia_ + Si ➔ SiC (9)

C_Dia_ + Ti ➔ TiC (10)

3C_Dia_ + 4Al_liq_ ➔ Al_4_C_3_  on (100) face [29] (11)

2C + 3Cr ➔ Cr_3_C_2_  [5] (12)

Significantly, different crystallographic planes of diamond feature different wettabilities. Aluminum was experimentally demonstrated [29,30] to wet well the {001} surface, the square facets of diamond, while not sticking to the hexagonal {111} facets. The values suggested for a typical thermal conductance of the diamond-aluminum interface are *G_001_* = 1 × 10^8^ W/(m^2^·K) and *G_111_* = 1 × 10^7^ W/(m^2^·K) [28] and *G_001_* = 1 × 10^8^ W/(m^2^·K) and *G_111_* = 2 × 10^7^ W/(m^2^·K) [101]. These results are corroborated by Chu *et al.* [31], a publication that demonstrates the possibility of formation of diffuse bonding between the aluminum and {111} diamond faces in Al(Si)-based composites fabricated by gas pressure infiltration.

Thermal conductance of 1 × 10^7^–1 × 10^8^ W/(m^2^·K) is characteristic not only of the Al-SiC-C_dia_ boundary but of the Cu-Cr_3_C_2_-C_dia_ interface as well (3.5 × 10^7^ W/(m^2^·K) for the thermal conductivity of the composite of 640 W/(m·K)) [5]. It was shown also that the Cr_3_C_2_ coating is ~100 nm thick, with its thermal conductivity estimated as 19 W/(m·K). To cite an example, Tavangar *et al.* [102] report 6 ± 1 × 10^7^ W/(m^2^·K) for thermal conductance of a 15-nm thick silver film on the (100) surface of synthetic diamond.

It is known that the linear thermal expansion coefficient of diamond is 1.18 × 10^–6^ K^-1^, and that of copper, 16.6 × 10^-6^ K^-1^. The coefficient of linear thermal expansion (CTE) of a composite is calculated from the equation (13) proposed by Turner [103]:

(13) $$\alpha_{c}=\frac{\alpha_{1}K_{1}\frac{F_{1}}{\rho_{1}}+\alpha_{2}K_{2}\frac{F_{2}}{\rho_{2}}+...}{K_{1}\frac{F_{1}}{\rho_{1}}+K_{2}\frac{F_{2}}{\rho_{2}}+...}$$

where *α*_i_ is the coefficient of volume thermal expansion of the i*th* component, *K_i_* is the bulk modulus of the i*th* component, *F_i_* is the weight fraction of the i*th* component, and *ρ_i_* is the density of the i*th* component.

Another model which can be used to calculate thermal expansion includes shear between the grains or phases and can be cast in the form (14) proposed by Kerner [104]:

(14) $$\alpha_{c}=\alpha_{1}+V_{2}(\alpha_{2}-\alpha_{1})\frac{K_{1}{\left(3K_{2}+4G_{1}\right)}^{2}+(K_{2}-K_{1})(16{G_{1}}^{2}+12G_{1}K_{2})}{\left(4G_{1}+3K_{2})[4V_{2}G_{1}(K_{2}-K_{1})+3K_{1}K_{2}+4G_{1}K_{1}\right]}$$

where *G_i_* are shear moduli of the i*th* phase.

**Figure 4:**
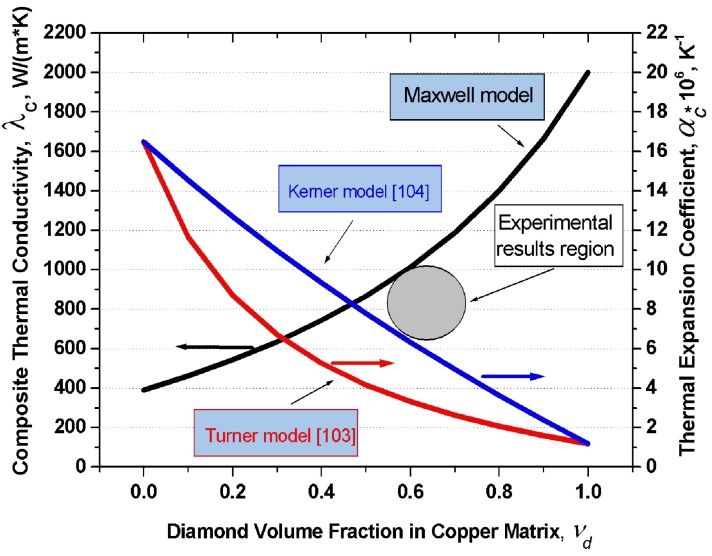
Graphs of the thermal conductivity in the diamond-copper system calculated in 16 the frame of the Maxwell model, and of the thermal expansion coefficient in terms of the 17 models of Turner and Kerner, plotted *vs*. composite composition. Grey circle indicates experimental values of the thermal conductivity and thermal expansion coefficient from [102].

Figure 4 displays graphically the dependence of thermal conductivity of the diamond-copper composite on diamond volume fraction which was calculated in terms of the Maxwell model under the assumption of the thermal conductivity of copper being 390 W/(m·K), and of that of diamond, 2,000 W/(m·K). Also plotted are the thermal expansion coefficients of the copper-diamond composite based on the model of Turner and Kerner. For the thermal expansion coefficients of copper and diamond one took *α*_Cu_ = 16.5 × 10^−6^ K^−1^ and *α*_dia_ = 1.2 × 10^−6^ K^−1^, respectively.

The values of *K* and *G* were taken from a handbook [105]: *K_Cu_* = 143 GPa, *K_dia_* = 500 GPa, *G_Cu_* = 48 GPa, *G_dia_* = 300 GPa. An analysis of this problem leads to a conclusion that in order to produce composites with the highest possible thermal conductivities, one should use silver, copper or aluminum as the matrix metal. Proper wettability of diamond by these metals can be ensured by growing on the diamond surface transition layers with carbide-forming elements. One conventionally uses for this purpose boron, chromium, titanium, tungsten, or silicon. The optimum thickness of these transition layers is confined to the 50–200 nm interval. Different diamond crystallographic planes react differently with carbide-forming elements. One should therefore choose diamonds which feature predominantly the desired crystallographic faces. The thermal conductivity of a composite can be enhanced by judiciously increasing the volume fraction of diamond in the composite, which, in its turn, can be increased by properly selecting the crystal habitus.

## 6. Composites Fabricated by Pulsed Plasma Sintering

Pulsed Plasma Sintering, a method developed in the 1960s and refined by Japanese scientists at the end of the XX century, made it possible to fabricate composite materials with novel characteristics in an approach based on passing high-power currents through the sample. This technique removes, in particular, the thin oxide layer present on practically all surfaces. Cleaned of the surface oxide layer, particles allow tighter packing which provides improved parameters of the interface separating the composite grains.

This approach is employed primarily in fabrication of ceramic materials and composites of refractory alloys-tungsten/cobalt alloys with the cobalt serving as the binder phase (WC/Co), ceramic or metal/ceramic cutting tools, corrosion- and wear-resistant materials. The SPS technique was found to be instrumental in sintering composites based on ceramic/metal and polymer/metal combinations, in development of tools fabricated by sintering cobalt and bronze compounds, crushed and bulk rocks, cutting blades, *etc*.

It was demonstrated, in particular, that the relative density of nitrogen-containing austenitic stainless steel compacted by spark plasma sintering (SPS) at different temperatures—1,073 K, 1,173 K, and 1,273 K in vacuum, at a pressure of 40 MPa and for a sintering time of 480 s, with particle size of 55 µm (powder A) and 27 µm (powder B), can be as high as 98.99% for A-series specimens and 99.78% for B-series ones [106].

There are, however, publications reporting on fabrication and study of high thermal conductivity composites as well. A few recent studies dealing with this problem [107,108] conclude that copper/diamond composites may be considered next-generation materials for heat sink applications. This has become possible due to the remarkable breakthrough in the development of an improved interface between copper and diamond, which is based, in particular, on vapor-phase deposition of chromium on the diamond surface. The thermal conductivities of these composites reached thus far are 284 W/(m·K), a figure exceeding by 30% those achieved with composite diamonds not coated by a metal.

## 7. Future Prospects and Current Problems in the Application of Metal/Diamond Composites as Heat Sink Materials

As already mentioned, the linear thermal expansion coefficient of diamond is 1.18 × 10^−6^ K^−1^, and that of copper, 16.6 × 10^−6^ K^−1^. It is desirable that the composition of the composite intended for use as heat sink material have a thermal expansion coefficient as close as possible to that of the semiconductor of interest. An ideal heat sink material should have a thermal expansion coefficient (CTE) from 3.5 × 10^−6^ K^−1^ to 6 × 10^−6^ K^−1^, an interval typical of the widely used semiconductors, such as Si, InP, GaAs, *etc*., and a high thermal conductivity.

Weber *et al.* [99] showed that adding to a diamond/copper composite boron with a concentration of 0.001 to 0.1 atom per copper atom brought the thermal expansion coefficient to the level of ~7 × 10^−6^ K^-1^, and for chromium added in a similar concentration it was 10 × 10^−6^ K^−1^.

The difficulties arising in an attempt to optimize the composition of a composite so as to reach a high thermal conductivity and a CTE consistent with that of semiconductors could be possibly resolved by adding a third or even a fourth component to the composite, provided that this would not result in a noticeable degradation of the thermal conductivity. The high electrical conductivity of such heat sinks, comparable with that of copper, would also pose a serious problem.

CVD-produced diamond plates may provide a serious competition to the heat sink materials based on diamond/copper composites. Industry has already produced by the CVD method samples of heat sinks with a thermal conductivity as high as, and even in excess of 1,000 W/(m·K), which measure 10 × 10 mm and are up to 1 mm thick. Their price, however, is presently fairly high.

## 8. Conclusions

The papers surveyed in the present review could hardly have been expected to cover all the aspects of the problem of fabrication of diamond-based composites; neither have we succeeded in discussing the achievements of all the research groups working in this area. No attention was focused on composites designed for development of materials for the industry manufacturing tools and abrasives.

We have, however, considered all the principal methods presently in use in fabrication of diamond-based composites, namely, infiltration, high pressure and temperature sintering, and spark plasma sintering, an approach which has lately been attracting considerable interest. An attempt has been undertaken to compare their merits and shortcomings.

Models were considered which are used to calculate the thermal conductivity of composites, from the Maxwell model to those taking into account the shape of particles. Particular attention was paid to the nanomaterial attracting considerable interest today - detonation nanodiamond, with a crystallite size of 5 nm, and physical aspects of the phase transitions occurring in this material were treated in comparison with microcrystalline diamond. Positive and negative aspects inherent in fabrication of composites based on detonation nanodiamond were analyzed.

The quality of the interface separating the particles making up a composite, a point we believe to be of major significance for development of high thermal conductivity composites, was studied. Solving the main problem, which lies in reducing the thermal conductance of the diamond/copper boundary, will significantly increase the thermal conductivity of the composites.

## References

[1] Slack G.A. (1973). Nonmetallic Crystals with High Thermal Conductivity. J. Phys. Chem. Solids.

[2] Lienhard J.H., Lienhard J.H. (2005). A Heat Transfer Textbook.

[3] Burgemeister E.A. (1977). Thermal Resistance at Metal/Diamond Interfaces in Relation to the Mounting of Microwave Diods. J. Phys. D: Appl. Phys..

[4] Sung C.M., Tai M.F. (1997). Reactivities of transition metals with carbon: Implications to the mechanism of diamond synthesis under high pressure. Int. J. Refract. Met. Hard Mater..

[5] Schubert T., Ciupiński Ł., Zieliński W., Michalski A., Weißgärber T., Kieback B. (2008). Interfacial characterization of Cu/diamond composites prepared by powder metallurgy for heat sink applications. Scr. Mater..

[6] Breval E., Cheng J., Agrawal D.K. (2000). Development of Titanium Coatings on Particulate Diamond. J. Am. Ceram. Soc..

[7] Hong S.M., Akaishi M., Yamaoka S. (1999). High-Pressure Synthesis of Heat-Resistant Diamond Composite Using a Diamond–TiC0.6 Powder Mixture. J. Am. Ceram. Soc..

[8] Ekimov E.A., Suetin N.V., Popovich A.F., Ralchenko V.G. (2008). Thermal Conductivity of Diamond Composites Sintered under High Pressures. Diamond. Relat. Mater..

[9] Kanda H. (2000). Large Diamonds Grown at High Pressure Conditions. Braz. J. Phys..

[10] Abbaschian R., Zhu H., Clarke C. (2005). High Pressure-High Temperature Growth of Diamond Crystals Using Split Sphere Apparatus. Diamond Relat. Mater..

[11] Itskevich E.S. (1997). High Pressure Physics. Soros Educ. J..

[12] Abyzov A.M., Kidalov S.V., Shakhov F.M. (2008). Composite Material of Diamond-Copper with High Thermal Conductivity. Mater. Sci. Trans. (Materialovedenie).

[13] Miyamoto Y., Kashiwagi T., Hirota K., Yamaguchi O., Moriguchi H., Tsuduki K., Ikegaya A. (2003). Fabrication of New Cemented Carbide Containing Diamond Coated with Nanometer-Sized SiC Particles. J. Am. Ceram. Soc..

[14] Swartz E.T., Pohl R.O. (1989). Thermal boundary resistance. Rev. Mod. Phys..

[15] Yoshida K., Morigami H. (2004). Thermal Properties of Diamond/Copper Composite Material. Microelectron. Reliab..

[16] Young D.A., Maris H.J. (1989). Lattice-Dynamical Calculation of the Kapitza Resistance between fcc Lattices. Phys. Rev. B.

[17] Stoner R.J., Maris H.J., Anthony T.R., Banholzer W.F. (1992). Measurements of the Kapitza Conductance between Diamond and Several Metals. Phys. Rev. Lett..

[18] Stoner R.J., Maris H.J. (1993). Kapitza Conductance and Heat Flow between Solids at Temperatures from 50 to 300 K. Phys. Rev. B.

[19] Kapitza P.L. (1941). The study of heat transfer in helium II. J. Phys. USSR.

[20] Weitz D.A. (2004). Packing in the Spheres. Science.

[21] Donev A., Cisse I., Sachs D., Variano E.A., Stillinger F.H., Connelly R., Torquato S., Chaikin P.M. (2004). Improving the Density of Jammed Disordered Packings Using Ellipsoids. Science.

[22] Wouterse A., Williams S.R., Philipse A.P. (2007). Effect of Particle Shape on the Density and Microstructure of Random Packings. J. Phys.: Condens. Matter.

[23] Liang X., Earthman J. C., Wolfenstine J., Lavernia E.J. (1992). A Comparison of Techniques for Determining the Volume Fraction of Particulates in Metal Matrix Composites. Mater. Charact..

[24] Bruggeman D.A.G. (1935). The Prediction of the Thermal Conductivity of Heterogeneous Mixtures. Ann. Phys..

[25] Wang J., Yi X.S. (2004). Effects of Interfacial Thermal Barrier Resistance and Particle Shape and Size on the Thermal Conductivity of AlN/PI Composites. Comput. Sci. Tech..

[26] Hasselman D.P.H., Johnson L.F. (1987). Effective Thermal Conductivity of Composites with Interfacial Thermal Barrier Resistance. J. Compos. Mater..

[27] Hamilton R.L., Crosser O.K. (1962). Thermal Conductivity of Heterogeneous Two-Component Systems. Ind. Eng. Chem. Fund..

[28] Flaquer J., Ríos A., Martín-Meizoso A., Nogales S., Böhm H. (2007). Effect of Diamond Shapes and Associated Thermal Boundary Resistance on Thermal Conductivity of Diamond-Based Composites. Comput. Mater. Sci..

[29] Kleiner S., Khalid F.A., Ruch P.W., Meier S., Beffort O. (2006). Effect of Diamond Crystallographic Orientation on Dissolution and Carbide Formation in Contact with Liquid Aluminium. Scr. Mater..

[30] Ruch P.W., Beffort O., Kleiner O., Weber L., Uggowitzer P.J. (2006). Selective Interfacial Bonding in Al(Si)–Diamond Composites and its Effect on Thermal Conductivity Compos. Sci. Technol..

[31] Chu K., Jia Ch., Liang X., Chen H., Gao W., Guo H. (2009). Modeling the Thermal Conductivity of Diamond Reinforced Aluminium Matrix Composites with Inhomogeneous Interfacial Conductance. J. Mater. Design.

[32] Yang H.S., Bai G.R., Thompson L.J., Eastman J.A. (2002). Interfacial Thermal Resistance in Nanocrystalline Yttriastabilized Zirconia. Acta Mater..

[33] Nan C.W., Birringer R., Clarke D.R., Gleiter H. (1997). Effective Thermal Conductivity of Particulate Composites with Interfacial Thermal Resistance. J. Appl. Phys..

[34] Angadi M.A., Watanabe T., Bodapati A., Xiao X., Auciello O., Carlisle J.A., Eastman J.A., Keblinski P., Schelling P.K., Phillpot S.R. (2006). Thermal Transport and Grain Boundary Conductance in Ultrananocrystalline Diamond Thin Films. J. Appl. Phys..

[35] Millett P.C., Wolf D., Desai T., Rokkam S., El-Azab A. (2008). Phase-Field Simulation of Thermal Conductivity in Porous Polycrystalline Microstructures. J. Appl. Phys..

[36] Prabu S.B., Karunamoorthy L., Kandasami G.S. (2004). A Finite Element Analysis Study of Micromechanical Interfacial Characteristics of Metal Matrix Composites. J. Mater. Process. Tech..

[37] Challis L.J. (1974). Kapitza Resistance and Acoustic Transmission across Boundaries at High Frequencies. J. Phys. C: Solid State Phys..

[38] Prasher R. (2009). Acoustic Mismatch Model for Thermal Contact Resistance of van der Waals Contacts. Appl. Phys. Lett..

[39] Duda J.C., Smoyer J.L., Norris P.M., Hopkins P.E. (2009). Extension of the Diffuse Mismatch Model for Thermal Boundary Conductance between Isotropic and Anisotropic Materials. Appl. Phys. Lett..

[40] Hopkins P.E. (2009). Multiple Phonon Processes Contributing to Inelastic Scattering During Thermal Boundary Conductance at Solid Interfaces. J. Appl. Phys..

[41] Reddy P., Castelino K., Majumda A. (2005). Diffuse Mismatch Model of Thermal Boundary Conductance Using Exact Phonon Dispersion. Appl. Phys. Lett..

[42] Cannon P. (1962). The Formation of Diamond. I. Demonstration of Atomic Processes Involving Carbon. J. Am. Chem. Soc..

[43] Kennedy C.S., Kennedy G.C. (1976). The Equilibrium Boundary between Graphite and Diamond. J. Geophys. Res..

[44] Bundy F.P., Bassett W.A., Weathers M.S., Hemley R.J., Mao H.K., Goncharov A.F. (1996). The Pressure-Temperature Phase and Transformation Diagram for Carbon. Carbon.

[45] Pope B.H., Horton M.D., Hall H.T., Divita S., Bowman L.S., Adaniya H.N. Sintered Diamond: Its Possible Use as a High Thermal Conductivity Semiconduction Device Substrate. Proceedings of the 4th International Conference on High Pressure (AIRAPT).

[46] Shulzhenko A.A., Gargin V.G. (1984). Properties and Structure of Diamond Polycrystals Obtained at Different Sintering Temperatures. Superhard Mater..

[47] Sokolina G.A., Bantsekov S.V., Fedoseev D.V., Afanasieva L.F., Ponizovskii L.Z. (1986). Electroconductivity of Sintered Diamond Powders. Superhard Mater..

[48] Akaishi M., Yamaoka S., Tanaka J., Ohsawa T., Fukunaga O. (1987). Synthesis of Sintered Diamond with High Electrical Resistivity and Hardness. J. Am. Ceram. Soc..

[49] Pantea C., Gubicza J., Ungar T., Voronin G.A., Zerda T.W. (2002). Dislocation Density and Graphitization of Diamond Crystals. Phys. Rev. B..

[50] Qian J., Pantea C., Voronin G., Zerda T.W. (2001). Partial Graphitization of Diamond Crystals under High-Pressure and High-Temperature Conditions. J. Appl. Phys..

[51] Voronov O.A., Kaurov A.A., Rakhmanina A.V. (1991). Properties of Natural Metamorphic Rock Diamond Crystals Compacts. Superhard Mater..

[52] Bochechka A.A., Romanko L.A., Gavrilova V.S., Konovalov S.M., Nazarchuk S.M. (2007). Special Features of Sintering Diamond Powders of Various Dispersions at High Pressures. Superhard Mater..

[53] Kidalov S.V., Shakhov F.M., Vul’ A.Ya. (2007). Thermal Conductivity of Nanocomposites Based on Diamonds and Nanodiamonds. Diamond Relat. Mater..

[54] Kidalov S.V., Shakhov F.M., Vul’ A.Ya. (2008). Thermal Conductivity of Sintered Nanodiamonds and Microdiamonds. Diamond Relat. Mater..

[55] Pantea C., Qian J., Voronin G.A., Zerda T.W. (2002). High Pressure Study of Graphitization of Diamond Crystals. J. Appl. Phys..

[56] Quin J., Pantea C., Huang J., Zerda T.W., Zhao Y. (2004). Graphitization of Diamond Powders of Different Sizes at High Pressure-High Temperature. Carbon.

[57] Kuznetsov V.L., Zilberberg I.L., Butenko Yu.V., Chuvilin A.L., Segall B. (1999). Theoretical Study of the Formation of Closed Curved Graphite-Like Structures During Annealing of Diamond Surface. J. Appl. Phys..

[58] Pantea C., Gubicza J., Ungar T., Voronin G.A., Nam N.H., Zerda T.W. (2004). High-Pressure Effect on Dislocation Density in Nanоsize Diamond Crystals. Diamond Relat. Mater..

[59] Belyankina A.V., Pugach E.A., Simkin E.S., Tsipin N.V. (1980). Phase Composition and Structure of Diamond Heatsink. Superhard Mater..

[60] Hansen M., Anderko K. (1958). Constitution of Binary Alloys.

[61] Sung J. (2000). Graphite ➔ Diamond Transition under High Pressure: A Kinetics Approach. J. Mater. Sci..

[62] Shulpiakov Yu.F., Dremin A.N., Doronin V.N., Zemlyakova L.G., Solovieva T.N. (1981). Distribution of Liquid Metal in Carbon at High Static Pressures. Inorg. Mater..

[63] Kanda H., Suzuki K., Fukunaga O., Setaka N. (1976). Growth of Polycrystalline Diamond. J. Mater. Sci..

[64] Akaishi M., Ohsawa T., Yamaoka S. (1991). Synthesis of Fine-Grained Polycrystalline Diamond Compact and Its Microstructure. J. Am. Ceram. Soc..

[65] Lin T.P., Hood M., Cooper G.A. (1994). Residual Stresses in Polycrystalline Diamond Compacts. J. Am. Ceram. Soc..

[66] Akaishi M., Kanda H., Sato Y., Setaka N., Ohsawa T., Fukunaga O. (1982). Sintering Behaviour of the Diamond-Cobalt Systems at High Temperature and Pressure. J. Mater. Sci..

[67] Ekimov E.A., Gierlotka S., Zibrov I.P., Gromnitskaya E.L., Presz A. (2004). Sintering of Diamond in the Presence of WO_3_. Inorg. Mater..

[68] Noma T., Sawaoka A. (1985). Fracture Toughness of High-Pressure-Sintered Diamond/Silicon Nitride Composites. J. Am. Ceram. Soc..

[69] Ko Y.S., Tsurumi T., Fukunaga O., Yano T. (2001). High Pressure Sintering of Diamonds-SiC Composite. J. Mater. Sci..

[70] Noma T., Sawaoka A. (1985). Effect of Heat Treatment of Fracture Toughness of Alumina-Diamond Composites Sintered at High Pressures. J. Am. Ceram. Soc..

[71] Wieligor M., Zerda T.W. (2008). Surface Stress Distribution in Diamond Crystals in Diamond–Silicon Carbide Composites. Diamond Relat. Mater..

[72] Pantea C., Voronin G.A., Zerda T.W. (2005). Kinetics of the Reaction Between Diamond and Silicon at High Pressure and Temperature. J. App. Phys..

[73] Gubicza J., Ungar T., Wang Y., Voronin G., Pantea C., Zerda T.W. (2006). Microstructure of Diamond–SiC Nanocomposites Determined by X-ray Line Profile Analysis. Diamond Relat. Mater..

[74] Pantea C., Voronin G.A., Zerda T.W., Zhang J., Wang L., Wang Y., Uchida T., Zhao Y. (2005). Kinetics of SiC formation during high P–T reaction between diamond and silicon. Diamond Relat. Mater..

[75] Voronin G.A., Zerda T.W., Gubicza J., Ungár T., Dub S.N. (2004). Properties of Nanostructured Diamond-Silicon Carbide Composites Sintered by High Pressure Infiltration Technique. J. Mater. Res..

[76] Ekimov E.A., Gromnitskaya E.L., Mazalov D.A., Pal’ A.F., Pichugin V.V., Gierlotka S., Palosz B., Kozubowski J.A. (2004). Microstructure and Mechanical Characteristics of Nanodiamond–SiC Compacts. Phys. Solid State.

[77] Ekimov E.A., Gromnitskaya E.L., Gierlotka S., Lojkowski W., Palosz B., Swiderska-Skoda A.A., Kozubowski J.A., Naletov A.M. (2002). Mechanical Behavior and Microstructure of Nanodiamond-Based Composite Materials. J. Mater. Sci. Lett..

[78] Aleksenskii A.E., Baidakova M.V., Vul’ A.Ya., Siklitskii V.I. (1999). The structure of diamond nanoclusters. Phys. Solid State.

[79] Kulakova I.I., Gubarevich T.M., Dolmatov V.Yu., Rudenko A.P. (2000). Chemical properties of detonation-synthesized ultradispersed diamonds. Superhard Mater..

[80] Vityaz P.A., Senyut V.T. (2004). Compaction of Nanodiamonds Produced under Detonation Conditions and Properties of Composite and Polycrystalline Materials Made on Their Basis. Phys. Solid State.

[81] Senyut V.T., Mosunov E.I. (2004). Physical-Mechanical Properties of Nanocrystalline Materials Based on Ultrafine-Dispersed Diamonds. Phys. Solid State.

[82] Bochechka A.A. (2004). Effect of Degassing on the Formation of Polycrystals from Diamond Nanopowders Produced by Detonation and Static Syntheses. Phys. Solid State.

[83] Yushin G.N., Osswald S., Padalko V.I., Bogatyreva G.P., Gogotsi Y. (2005). Effect of Sintering on Structure of Nanodiamonds. Diamond Relat. Mater..

[84] Davydov V.A., Rakhmanina A.V., Agafonov V.N., Khabashesku V.N. (2007). Size-Dependent Nanodiamond-Graphite Phase Transition at 8 GPa. J. Exp. Theor. Phys. Letter..

[85] Oleynik G.S., Kotko A.V. (2008). Self-Organization of Ultradisperse Diamond Particles Heated at High Pressures. Tech. Phys. Lett..

[86] Aleksenskii A.E., Baidakova M.V., Vul’ A.Ya., Davydov V.Yu., Pevtsova Yu.A. (1997). Diamond-graphite phase transition in ultradisperse-diamond clusters. Phys. Solid State.

[87] Chen J., Deng S.Z., Chen J., Yu Z.X., Xu N.S. (1999). Graphitization of Nanodiamond Powder Annealed in Argon Ambient. Appl. Phys. Lett..

[88] Andreev V.D. (1999). Spontaneous graphitization and thermal disintegration of diamond at T > 2000 K. Phys. Solid State.

[89] Kuznetsov V.L., Butenko V.L., Gruen D.M., Shenderova O.A., Vul’ A.Ya. (2005). Nanodiamond graphitization and properties of onion-like carbon. Proceedings of the NATO Advanced Research Workshop on Ultrananocrystalline Diamond, St. Petersburg, Russia, June 2004; Synthesis, Properties and Applications of Ultrananocrystalline Diamond.

[90] Zhao D.S., Zhao M., Jiang Q. (2002). Size and Temperature Dependence of Nanodiamond-Nanographite Transition Related with Surface Stress. Diamond Relat. Mater..

[91] Jiang Q., Li J.C., Wilde G. (2000). The Size Dependence of the Diamond–Graphite Transition. J. Phys.: Condens. Mater..

[92] Dubrovinskaia N., Dubrovinsky L., Langenhorst F., Jacobsen S., Liebske C. (2005). Nanocrystalline Diamond Synthesized from C_60_. Diamond Relat. Mater..

[93] Yang C.C., Li S. (2008). Size-Dependent Temperature-Pressure Phase Diagram of Carbon. J. Phys. Chem. C.

[94] Shulzhenko A.A., Bochechka A.A., Romanko L.A., Kutsay A.M., Gargin V.G. (2000). Peculiarities of sintering of vacuum heat-treated nanometric diamond powders. Superhard Mater..

[95] Osswald S., Havel M., Mochalin V., Yushin G., Gogotsi Y. (2008). Increase of nanodiamond crystal size by selective oxidation. Diam. Rel. Mater..

[96] Obraztsov A.N., Pavlovsky I.Yu., Ralchenko I.Yu., Okushi H., Watanabe H. (1999). Determination of thermal conductivity of CVD diamond films via photoacoustic measurements. Appl. Phys. A: Mater. Sci. Process..

[97] Leung K.M., Cheung A.C., Liu B.C., Woo H.K., Sun C., Shi X.Q., Lee S.T. (1999). Measuring Thermal Conductivity of CVD Diamond and Diamond-Like Films on Silicon Substrates by Holographic Interferometry. Diamond Relat. Mater..

[98] Zhong Z., Wang X. (2006). Thermal Transport in Nanocrystalline Materials. J. Appl. Phys..

[99] Weber L., Tavangar R. (2007). On the Influence of Active Element Content on the Thermal Conductivity and Thermal Expansion of Cu–X (X = Cr, B) Diamond Composites. Scr. Mater..

[100] Molina J.-M., Rhême M., Carron J., Weber L. (2008). Thermal Conductivity of Aluminum Matrix Composites Reinforced with Mixtures of Diamond and SiC Particles. Scr. Mater..

[101] Böhm H.J., Nogales S. (2008). Mori–Tanaka Models for the Thermal Conductivity of Composites with Interfacial Resistance and Particle Size Distributions. Compos. Sci. Technol..

[102] Tavangar R., Molina J.M., Weber L. (2007). Assessing Predictive Schemes for Thermal Conductivity Against Diamond-Reinforced Silver Matrix Composites at Intermediate Phase Contrast. Scr. Mater..

[103] Turner P.S. (1946). Thermal-Expansion Stresses in Reinforced Plastics. J. Res. NBS.

[104] Kerner E.H. (1956). The Elastic and Thermoelastic Properties of Composite Media. Proc. Phys. Soc..

[105] Cardarelli F. (2008). Materials Handbook. A Concise Desktop Reference.

[106] Xu Z., Jia Ch., Kuang Ch., Chu K., Qu X. (2009). Spark Plasma Sintering of Nitrogen-Containing Nickel-Free Stainless Steel Powders and Effect of Sintering Temperature. J. Alloys Compd..

[107] Chu K., Liu Zh., Jia Ch., Chen H., Liang X., Gao W., Tian W., Guo H. Thermal Conductivity of SPS Consolidated Cu/Diamond Composites With Cr-Coated Diamond Particles. J. Alloys Compd..

[108] Chu K., Jia Ch., Tian W., Liang X., Chen H., Guo H. Thermal Conductivity of Spark Plasma Sintering Consolidated SiCp/Al Composites Containing Pores: Numerical Study and Experimental Validation. Compos. Part A: Appl. Sci. Manuf..

